# Clinical Predictors of Mortality in Adults with Intellectual Disabilities with and without Down Syndrome

**DOI:** 10.1155/2012/943890

**Published:** 2012-05-16

**Authors:** Lilian Thorpe, Punam Pahwa, Vernon Bennett, Andrew Kirk, Josephine Nanson

**Affiliations:** ^1^Departments of Community Health & Epidemiology and Psychiatry, College of Medicine, University of Saskatchewan, Saskatoon, SK, Canada S7N 0W8; ^2^Department of Psychiatry, College of Medicine, University of Saskatchewan, Saskatoon, SK, Canada S7N 0W8; ^3^Division of Neurology, Department of Medicine, University of Saskatchewan, Saskatoon, SK, Canada S7N 0W8; ^4^Department of Psychology, University of Saskatchewan, Saskatoon, SK, Canada S7N 5A5

## Abstract

*Background.* Mood, baseline functioning, and cognitive abilities as well as psychotropic medications may contribute to mortality in adults with and without Down Syndrome (DS). *Methods.* Population-based (nonclinical), community-dwelling adults with intellectual disabilities (IDs) were recruited between 1995 and 2000, assessed individually for 1–4 times, and then followed by yearly phone calls. *Results.* 360 participants (116 with DS and 244 without DS) were followed for an average of 12.9 years (range 0–16.1 years as of July 2011). 108 people died during the course of the followup, 65 males (31.9% of all male participants) and 43 females (27.6% of all female participants). Cox proportional hazards modeling showed that baseline practical skills, seizures, anticonvulsant use, depressive symptoms, and cognitive decline over the first six years all significantly contributed to mortality, as did a diagnosis of DS, male gender, and higher age at study entry. Analysis stratified by DS showed interesting differences in mortality predictors. *Conclusion.* Although adults with DS have had considerable improvements in life expectancy over time, they are still disadvantaged compared to adults with ID without DS. Recognition of potentially modifiable factors such as depression may decrease this risk.

## 1. Introduction

Although much improvement has occurred during the last century, mortality rates in people with childhood onset intellectual disabilities (IDs) are still higher than those of the general population, especially in younger adults in their 20s and people with Down syndrome (DS) [1]. In general populations, increased age is known to be an important predictor of increased mortality, as is male gender, although some data suggests that males with DS may have a relative survival advantage [2]. In general, mortality rates are lower in community samples, although this may not be true for those with severe disabilities, whose needs may be met less well in the community [3, 4].

Also of potential significance to mortality is the Intelligence Quotient (IQ). Among people with ID, those with the most severe impairment were found in Patja et al's cohort study (previously referenced) to have significantly lower life expectancy, whereas those with mild ID had similar life expectancy to the general population. This difference in life expectancy is likely related to increased severity of underlying medical illness in those with the greatest intellectual impairment.

In the general population, excess mortality (especially due to cardiac and respiratory diseases) has been found in those with major mental illness [5], case level depression [6] and those who have depressive symptoms and medical illness such as unstable angina [7]. Depressive symptoms have been linked to decreased total active (and total) life expectancy [8, 9], with some suggestions by Win et al. that some of this is mediated by physical inactivity. Other reports have linked autonomic dysfunction and inflammation to the increased cardiovascular mortality risk associated with depression [10]. Depressive symptoms have also been linked to an increased risk of dementia in the general population [11] and in a DS population [12], but reasons for this association have not fully been clarified. It is possible that depression is itself a very early manifestation of the development of a degenerative process such as Alzheimer's disease, but it may also exert (directly or indirectly) adverse effects on the biological structures in the brain, causing or accelerating the degenerative process of dementia (although recent neuropathological work by Tsopelas et al. [13] makes the latter explanation less likely). Dementia in turn has been associated with increased mortality in the general population [14].

Other potential contributors to mortality include the use of psychotropic medications. There has been particular concern about the use of antipsychotic medications in people with dementia, with some studies (but not all) suggesting increased mortality and strokes (see review by [15]). Associations between antipsychotics and adverse health outcomes are clearly not specific to people with dementia, as shown in general population studies of increased sudden cardiac death related to antipsychotics [16]. Adults with ID are commonly prescribed antipsychotics [17–20], often for behavioural problems, and may therefore be particularly impacted by this adverse outcome.

Anticonvulsants are another potential contributor to increased mortality. Although epilepsy itself is associated with increased mortality including sudden unexplained death [21–23] and new onset seizures are thought to be markers for cognitive decline in people with DS (see review in [24]), recent epidemiologic and placebo-controlled trials data suggest that increased rates of death, especially violent death including suicides, may be related to anticonvulsants themselves [25, 26]. As anticonvulsants are used frequently in people with ID, who have a high rate of epilepsy as well as behavioral problems for which anticonvulsants are used, this might be an important and potentially modifiable factor in improving mortality rates.

The population-based (nonclinical), Intellectual Disability and Aging Study was designed in the early 1990s to fill gaps in clinical understanding of longitudinal cognitive and functional changes as well as mortality patterns in adults with Down Syndrome (DS). After the methodology was discussed at various university and community forums, adults with ID but not DS were added to the study to provide an appropriate control group. This paper focuses particularly on baseline contributors to mortality.

## 2. Methods

 Appropriate authorization for whole study was obtained from the University of Saskatchewan Ethics Committee. Procedures followed were in accordance with the ethical standards of the Helsinki Declaration of 1975, as revised in 2000 [27]. Letters requesting participation of any adult with caregiver (or family) defined childhood onset intellectual disability aged 18 years and over were sent in 1995 to all provincial community services (group homes, independent living organizations, supportive work settings) designated for adults with ID. There were no additional exclusion criteria. Information was provided about the study, and clinical coordinating staff was asked to forward enclosed consent forms to potential participants or their usual substitute decisionmakers for medical decisions. Participants who clearly understood the process of the study were asked to provide their own consent. If the potential participant assented but obviously lacked capacity for full, informed consent, the person who normally consented to health care interventions provided consent. If there was assent but partial or unclear capacity to consent, both the person and their usual medical decisionmaker would provide consent. No participants were included whose family or immediate caregivers voiced opposition to participation after full information was provided. Participants or their substitute decisionmakers mailed signed consent forms back in provided envelopes. Research staff made periodic phone contact as necessary with community service providers to provide further information about the study and to answer questions about eligibility and appropriate provision of consent. One exception was made to accept a 17 year old participant whose substitute decision-maker mailed in a consent form.

Once completed consent forms were received, questionnaires addressing basic demographics, residential information (type of living situation), name of family physician, psychiatric care, name of social services case manager, basic health information (including suspected or confirmed dementia), seizure history and frequency of current seizures, medication use, estimated level of disability (profound, severe, moderate, mild, and borderline), and most recent IQ score before the age of 18 were mailed to the care provider who was most familiar with the participant. Care providers were also mailed copies of standardized caregiver-rated instruments (described in the following) to complete. After receiving the completed questionnaires and instruments, research staff contacted care providers to assess the participant's ability and/or willingness to engage in direct interviews and testing. 276 participants eventually had at least one direct assessment consisting of a variety of instruments, which are not described here as they were not used in the analysis forming the basis of this paper. All information was reviewed by the primary investigator, and additional contacts were made with care staff, families, and medical staff as necessary to confirm accuracy of information.

To establish to representativeness of our initial sample, we obtained baseline 1995 service provision data (numbers and age distribution) from the division of the Department of Social Services responsible for people with ID, as our sample was drawn from people participating in those services.

No financial reimbursement was given to participants, but at each wave of direct data collection a printed certificate of participation was presented.

### 2.1. Data Collection

Formal instruments used in the study were chosen for their ease of administration, acceptability, validity, and psychometric data and had to be further amended by the funding and manpower available. Final instruments included in the caregiver package were Evenhuis' Dementia Questionnaire for Persons with Mental Retardation (DMR: [28]) and the Reiss Screen for Maladaptive Behavior [29].

### 2.2. Dementia Questionnaire for Persons with Mental Retardation (DMR)

The Evenhuis questionnaire was chosen for the evaluation of cognitive and functional decline in those with ID as it was one of the practical and well-known caregiver-rated instruments designed for this purpose, and our study did not have resources to provide comprehensive individualized dementia diagnoses to participants across the province. The standardized 50-item instrument is based on the dementia criteria in the DSMIII-R [30] but was adapted to allow for easier scoring in those with baseline intellectual disabilities. Higher scores on the DMR (based on behaviour over the last three months) indicate more impairment. Subscales of the DMR include short-term memory, long-term memory, spatial and temporal orientation, speech, practical skills, mood, activity and interest and behavioural disturbance.

The DMR subscales themselves have been summed to derive two major subscales: the Sum of Cognitive Scores (SCS: short-term memory, long-term memory, spatial and temporal orientation), which have a score range of 0 to 44, and the Sum of Social Scores (SOS: speech, practical skills, mood, activity and interest and behavioral disturbance), which has a range of 0 to 60. The preferred use of the DMR in the screening for dementia is by analyzing longitudinal score changes, as the baseline IQ affects most of the items in the DMR. Evenhuis' published criterion for a positive dementia screen on the basis of longitudinal score changes is either an increase of the SCS of 7 points or more and/or an increase of the SOS of 5 points or more over subsequent tests.

Manpower was not available to provide individual medical assessments and diagnosis of cognitive impairment and/or dementia. We therefore decided to use individual measures of yearly decline on the SCS and the SOS from scores on the first four detailed assessments for each participant in the study, using the least squares method [31]. This results in a separate slope for each individual representing change over time in each subscale. The formula used to derive the slope is shown as follows

(1) $${\text{Slope}}_{i}=\frac{\sum_{j=1}^{4}(x_{ij}-\overset{̅}{x_{i}})\left(y_{ij}-\overset{̅}{y_{i}}\right)}{\sum_{j=1}^{4}{\left(x_{ij}-\overset{̅}{x_{i}}\right)}^{2}}.$$

In this equation, for *n* participants who had 4 tests each, *y*_*ij*_ represents the outcome for the *i*th participant at the *j*th time, and *x*_*ij*_ is the independent variable for the *i*th participant at the jth time. $\overset{̅}{y_{\iota }}$ represents the mean outcome for the *i*th participant, and $\overset{̅}{x_{\iota }}$ represents the mean value of the independent variable for the *i*th participant. We were aware that slopes derived with this method would capture only individual changes pooled over all of their first four assessments, rather than individual changes between specific assessments, and felt that this was a reasonable approach as individuals occasionally had fluctuations in their functioning in specific tests due to medical or social reasons.

### 2.3. The Reiss Screen for Maladaptive Behaviour (RSMB)

The 38-item Reiss Screen for Maladaptive Behavior [29] was chosen to screen for depressive symptoms because it was a well-known, caregiver-rated scale for people with ID (not necessarily old), whose scores on its various subscales could be compared to normative data, and correlated with psychiatric syndromes of clinical interest (in this case depression). The eight core psychiatric subscales of the Reiss Scale include aggressive behavior, autism, psychosis, paranoia, depression (behavioral signs: anxious, crying spells, fearful, overly sensitive, sadness), depression (physical signs: body stress, eating problem, low energy, regressive behaviour, sleep problem), dependent personality disorder, and avoidant personality disorder. Items were initially designed to be completed by two separate caregivers who know the person well, and final scores on each category were to be based on the average of the two scores. In clinical practice, the Reiss Screen is frequently completed by one caregiver because of time constraints. Scores above the published cutoff scores for the individual subscales (aggression:5, autism:4, psychosis:5, paranoia:5, depression (behavioral signs):5, depression (physical signs):4, dependent personality disorder:6, and avoidant personality disorder:5) indicate clinical problems and the need for a further clinical assessment. Good psychometric properties were described by Reiss et al. in [29], although abnormal scores in subscales are clearly not analogous to standard clinical diagnoses. Some concerns have more recently been expressed about the characteristics of many screening instruments, including the Reiss Screen, in people with ID [32]. However, at the time this study was conceived, it was not possible to administer more detailed or comprehensive assessments.

### 2.4. Data Management and Analysis

Full data were collected from four formal data-assessment waves (1995-1996, 1997, 1999, and 2001), and limited data (mortality, nursing home placement) was collected from ongoing follow-up telephone surveys, most recently in July 2011. All data were entered by research assistants into a secure access database designed by the principal investigator, and data accuracy was verified for at least 25% of all data entries in each wave. Data in one wave was reentered due to greater than 5% errors. Descriptive results of the data were initially organized into tabular and graphic forms, exploring the patterns of univariate associations between baseline variables including age, sex, DS diagnosis, seizure history, and frequency of current seizures, health problems (including baseline caregiver identified confirmed or suspected dementia), baseline cognitive and psychiatric symptoms (from the DMR and the Reiss Screen), psychotropic medication use, IQ score, and mortality. IQ was dropped from the analysis because not enough valid scores were available in the sample. The baseline DMR practical skills subscale score was chosen as the main measure approximating the level of global deficits at entry to the study, acknowledging that this level of baseline skills would represent both baseline adult abilities as well as decrements from any preclinical degenerative processes. Level of ID (borderline, mild, moderate, severe, and profound) was also dropped from the analysis because the interrater agreement across waves was low.

The remote history of seizures may not have been as accurate as information pertaining to seizures in the recent year, as there is known to be a high turnover in care staff, and many of our informants may have had mostly recent information about the participants. However, caregivers during the detailed assessments in waves 1 to 4 were asked to provide their best answers to the current and past presence of seizures based on all information available to them using the following rating: 0—never a history of seizures, 1—previously seizures but no seizures in the past year or more, 2—seizures occurring at the rate of less than one seizure per month, 3—one to four seizures per month, 4—two to six seizures per week, or 5—daily seizures. Seizures were explored in the regression analysis using a number of recoded variables: no seizures ever documented, seizures present at or before baseline, active seizures present at baseline, seizures reported in any of the four active data collection waves and seizures occurring during the followup but not at baseline (new seizures).

### 2.5. Statistical Analysis

Survival analysis was used to assess differential mortality during the course of the study. Participants were followed for a maximum of 16 years, with some (very few) leaving the study prematurely and some dying prior to the most recent contact in July 2011. Cox's proportional hazards modeling technique [33] was used to assess differential mortality, as it allows for the analysis of mortality rates based on different lengths of followup, adjusting for various independent variables in the regression model. It was not possible to do a time-dependent analysis for the independent variables as detailed data on most (such as medication use, occurrence of seizures) was only available for four waves of data collection, as described above. To compensate for this shortcoming, we used both the variable score at baseline and a recoded variable representing a pooled measure of the variable. For example, baseline use of antipsychotic medications was added into the model as well as a variable coding for the presence of an antipsychotic during any of the four detailed data collection waves. In the case of seizures, where the new onset of seizures is known to be associated with dementia in persons with DS, a third variable was created to represent people who developed new seizures after baseline.

Variables that were added to the initial Cox regression model using SPSS version 19 [34] included DS (0,1), sex (males = 1, females = 2), age (at baseline in years), number of years followed, deceased as of July 2011 (0,1), DMR practical skills subscale score (score 0–16), DMR Sum of Social Scores change per year and DMR Sum of Cognitive Scores individual change per year over waves 1–4, use of medications (antipsychotics, anxiolytics, antidepressants, sedative-hypnotics, anticonvulsants) at any of the four detailed waves of data collection, seizure status as described earlier, and the depression-related Reiss subscale scores at baseline (depression—behavioral, depression—physical).

Core variables based on DS diagnosis, age, sex, and DS-Age interaction term were kept in every initial model regardless of statistical significance. Terms were then removed manually from the model in order of least statistical significance. Results are presented as hazard ratios and their 95% confidence intervals. The proportionality assumption was satisfied when tested using the log minus log test.

## 3. Results

Participants came from all areas of the province except for the far north, with the largest number originating from urban centres, consistent with the population distribution of the province. Participant living situations included Community Living Division group homes, private care homes, mental health approved homes, assisted living facilities, independent dwellings, family homes, and one larger congregate living site (but not the provincial institution for ID).

This study population represented a sizable proportion of the overall service population recorded by the Department of Social Services in 1995. In 1995, 3214 people with ID received services or 0.32% of the total population of Saskatchewan based on the 1996 census. Our participants represented 9.9%, 22.8%, 17.4%, and 9% of this ID service population within age groups 21–35, 36–54, 55–64, and 65+, respectively.

**Figure 1:**
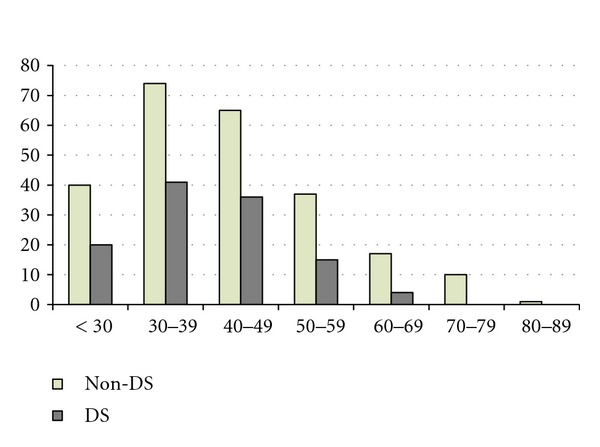
Age distribution of study participants at baseline (*N* = 360).

The 360 participants providing data between 1995 and 2011 included 142 non-DS males, 102 non-DS females, 64 DS males, and 52 DS females. More males than females entered the study (female to male ratio: 1 : 1.34), and the DS group was about three years younger on average than the non-DS group (*P* < 0.05 using independent samples *t*-test). Males and females were not significantly different in age. Basic demographics are shown in Table 1 and shown graphically in Figure 1.

Baseline scores on key variables entered into the initial model are shown in Tables 2 and 3.

**Figure 2:**
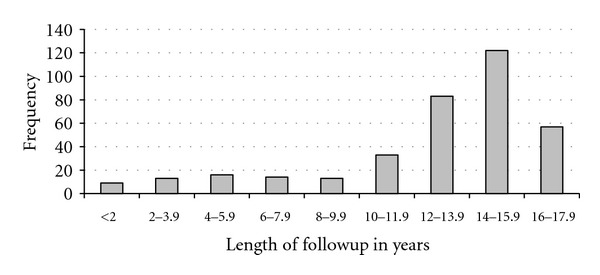
Length of follow-up of study participants by July 2011.

At the most recent analysis in July 2011, 108 participants had died, and 9 had withdrawn for various reasons, leaving 243 in active followup. Follow-up time varied from 0 (only one assessment before leaving the study for any reason) to 16.1 years as of July 2011, with the mean of 12.93 (0.21) years (Figure 2).

The number and percentages of deceased participants and mean ages of death and various categories are shown in Table 4.

Based on Cox proportional hazards models with the pooled DS and non-DS participants, leaving in the almost significant (*P* = 0.08) DS-age interaction term, sex, age at baseline, baseline practical skills deficits, baseline depression symptoms (Reiss behavioural depression), yearly decline on DMR social skills, a seizure history at baseline, a seizure history at any point before and during the study, and anticonvulsant use at baseline were all independently statistically significant to the prediction of mortality, as shown in Table 5. The derived seizure variable, new seizure, representing seizures arising after the beginning of the study, was not significant to mortality prediction. The use (baseline or during any of the first four waves) of psychotropic medications including antipsychotics, antidepressants, anxiolytics, and sedative-hypnotics were also not significant predictors of mortality. Also not significant was the caregiver designation of suspected or confirmed dementia or cognitive impairment at baseline.

A separate survival analysis (adjusting for DS, age, and sex) was performed to explore whether the use of an anticonvulsant during any of the first four detailed data collection waves in the absence of a current or previous history of seizures increased mortality. Although there was a trend supporting this, it was not significant (*P* = 0.26). Similarly, a separate survival analysis (adjusting for DS, age, and sex) was conducted to explore the possibility that the use of an antipsychotic in the absence of a clinical diagnosis reported by a caregiver of a mental health problem for which the use of an antipsychotic is generally appropriate (any psychotic disorder such as schizophrenia or delusional disorder, or bipolar disorder) might increase mortality. This was also negative (*P* = 0.25).

Because people with DS are known to have shorter lifespans and higher risks of dementia, even though the DS-Age interaction was not quite significant, the following analyses were repeated with the groups stratified by DS.  Table 6 shows that mortality in people without DS was predicted by increased age, higher levels of Reiss physical depression scores, greater decline in DMR social scores per year during the first four waves, and a seizure history at or before baseline. Unlike in the pooled DS and non-DS analysis, sex, baseline practical skills deficits, baseline anticonvulsant use, baseline Reiss behavioral depression, and seizure at any time before or during the study were not significant predictors of mortality.

Cox regression for mortality in participants with DS resulted in a different model.  Table 7 shows that mortality in people with DS was increased by male sex, older baseline age, increased baseline practical skills deficits, higher levels of baseline Reiss behavioural depression scores, and greater decline in DMR cognitive scores per year during the first four waves.

**Figure 3:**
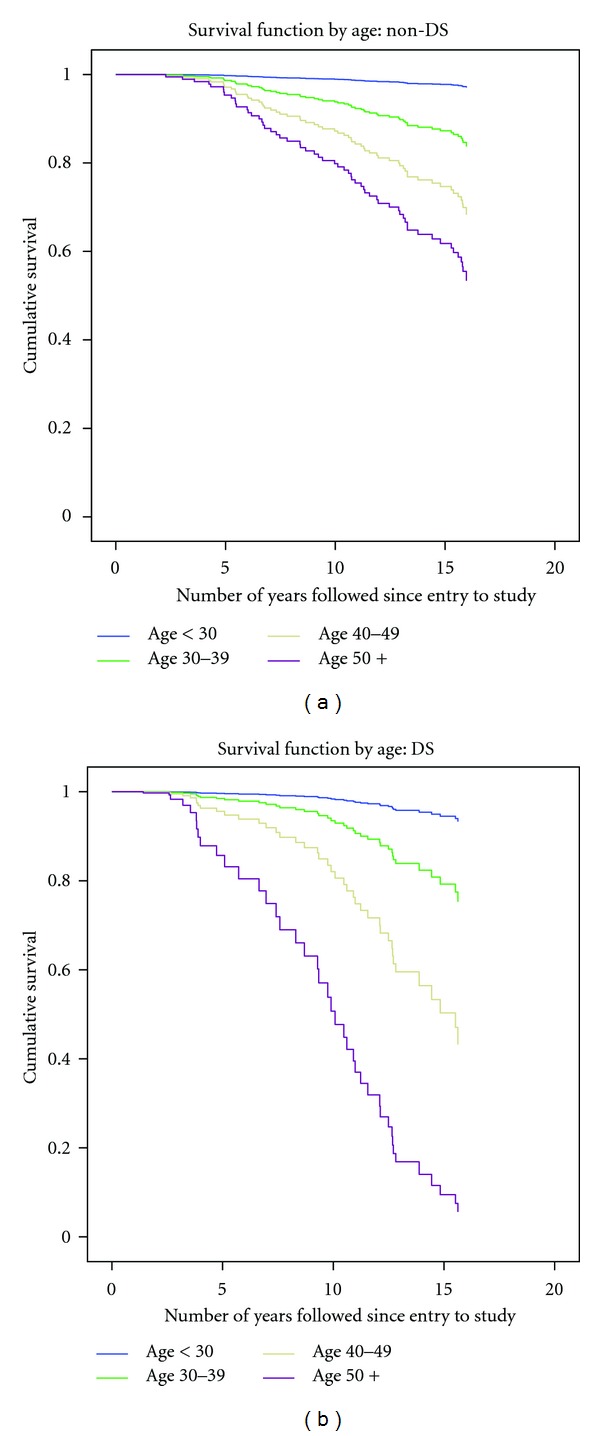
Adjusted survival curves of 360 participants with ID (1995–2011): impact of baseline age: non-DS compared to DS.

**Figure 4:**
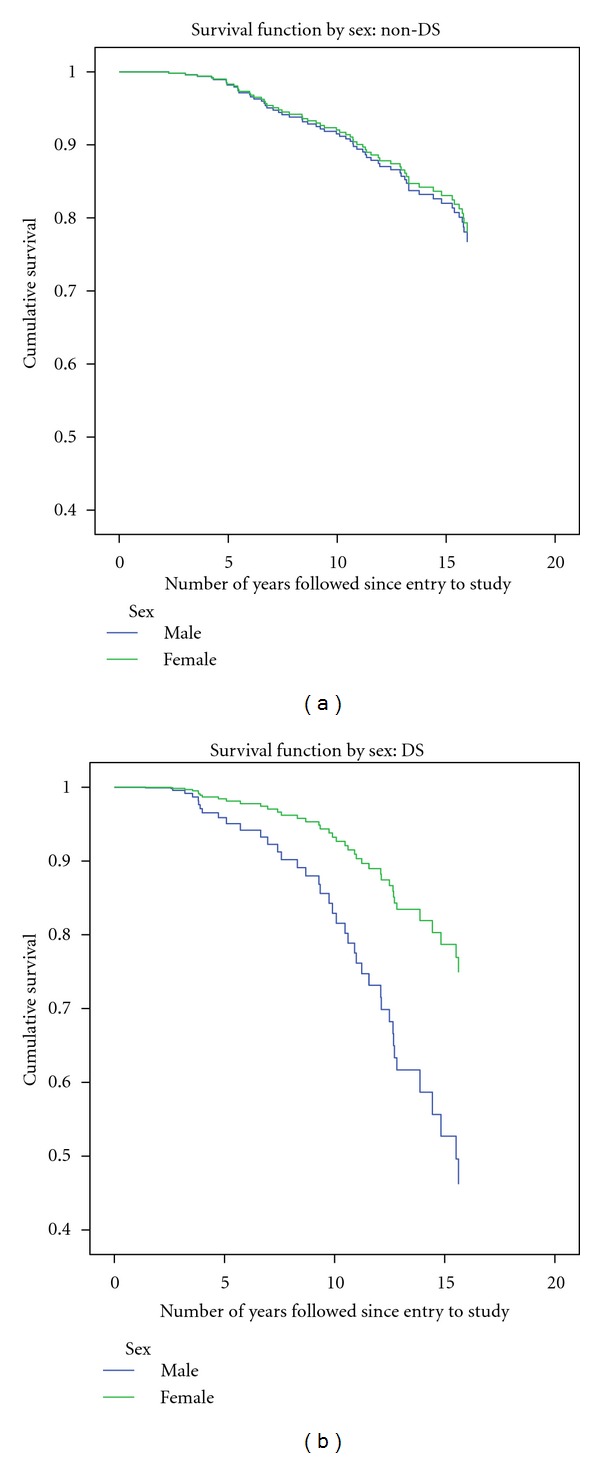
Adjusted survival curves of 360 participants with ID (1995–2011): impact of sex: non-DS compared to DS.

**Figure 5:**
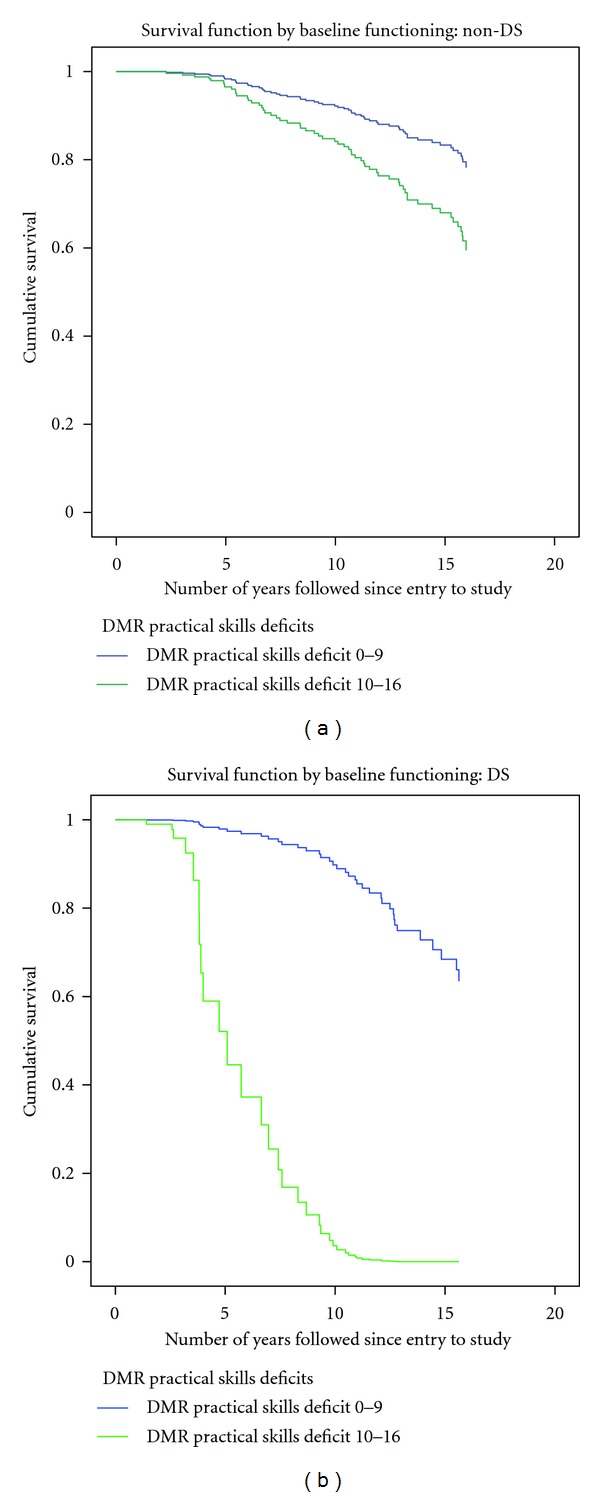
Adjusted survival curves of 360 participants with ID (1995–2011): impact of baseline functional deficits: non-DS compared to DS.

Contrasts between some of the survival curves (adjusted as shown in Tables 6 and 7) for participants with and without DS are illustrated in Figures 3, 4, and 5.

## 4. Discussion

### 4.1. Pooled DS and Non-DS Analysis

Based on our pooled DS and non-DS analysis (including the almost significant DS ∗ age interaction term), some of our findings regarding the prediction of increased mortality, such as the presence of DS, older age, and lower baseline level of baseline functioning, do not challenge the general understanding about mortality in ID. However, we did not find that males with DS had any special protection compared to females, unlike some findings reported by others (cited earlier). We instead found that males, just as in the general population, had increased mortality when findings were adjusted for the other significant predictors including age and baseline functioning.

We had not expected any impact of baseline depressive symptoms on the eventual mortality when the study was designed, and this association was found only after comprehensive exploration of all valid baseline variables in the Cox regression analysis. The ID population is not known to have high suicide rates, and especially in supportive settings in our community their level of preventive health care (and rates of smoking) is likely better than that in the general population. For example, most of our local community group homes have excellent policies regarding yearly medical assessments and screening. Self-harm attempts in people with depression related to driving (inattention, purposeful risk-taking) are also very unlikely in ID. The association between baseline depressive symptoms and increased mortality in our study is therefore consistent with previously cited data suggesting that some other factors, such as inactivity, autonomic dysfunction, and inflammation, may have an important role to play. It is also possible that depressive symptoms are a marker for early dementia, which is independently related to increased mortality. However, the risk remained even when adjusted for by yearly decline in social scores, as well as caregiver direct reports of dementia or other cognitive functional decline, so it would appear that depressive symptoms may still have an additive adverse impact.

In our study, the use of antidepressants at baseline or at any point in the first four data collection waves did not contribute significantly in either direction to mortality. Unfortunately, our data did not allow us to fully explore whether the treatment of depression with antidepressants (using a time covarying analytic technique) improved this increased mortality risk, as we did not have data on antidepressant use for the entire study period. This would have been more ideal, as the data on treatment of depression and mortality is contradictory, with some recent research, such as that from the Women's Health Initiative Study [35] even suggesting an increased mortality and stroke risk in women on these mediations.

The baseline use of anticonvulsants was associated with increased mortality in our sample, even when adjusted for seizures present at or before baseline and seizures present at any time during the study. Anticonvulsants have many adverse effects, including drug-drug interactions, which may have played a role. It would have been ideal to have detailed information about the time of the original onset of seizures, as this might have been significant to eventual mortality outcome. Although we did not find that there was an additional adverse impact on mortality by the use of anticonvulsants in the absence of seizures, the potential red flag of the use of anticonvulsants suggests increased caution in using these drugs, especially for behavioral reasons, where alternate interventions might be instituted.

The presence of a seizure before or at baseline independently increased mortality risk (*P* < 0.05), as did the presence of a seizure at any point during the study (*P* < 0.05). The significance of both of these predictors independently in the model may suggest that both an early onset of seizures and later seizures may have different mechanisms of action accounting for their association with increased mortality, but result in increased cumulative burden. For example, early onset epilepsy is associated in sudden unexplained death (cited earlier), and is also associated with other physical problems which may increase mortality, whereas later onset of seizures, especially in those with DS, is associated with the development of dementia, itself a predictor of increased mortality.

We did not find that the baseline use (or use during any of the first four waves of detailed data collection) of antipsychotics contributed to mortality, unlike some studies in frail, demented people without ID. It is possible that healthier, strong, people with ID may be more likely to exhibit risk to others from aggressive behaviours and are therefore more likely to be prescribed these agents. The prescription of antipsychotics then might be a marker for decreased mortality at baseline, masking any other direct adverse effects. We also did not find an increased adverse impact of the use of antipsychotics in the absence of a diagnosis of a psychotic or bipolar disorder, for which antipsychotic use is frequently indicated. Unfortunately, these diagnoses were obtained from caregivers and chart information rather than individual, standardized assessments, so their validity may be questionable.

Greater individual yearly changes in the DMR Sum of Social Scores over the first four data collection waves were associated with increased mortality, even when adjusted for by age and other factors. Although the yearly changes were very small in all groups, this association suggests that, even in young people, subtle decline in functioning might be predictive of later, poor outcomes.

### 4.2. Stratified DS and Non-DS Analysis

Stratification of the analysis into participants with and without DS had face value in light of the aging differences well established by others, although the reduction in numbers likely resulted in the loss of ability to find significance in some of the potentially predictive factors for mortality. For example, the use of anticonvulsants was no longer statistically significant to outcome in either group, sex was not significant to mortality in those without DS, and the impact of seizures in those with DS (low numbers) could not be ascertained. However, some patterns more specific to DS likely emerged from this approach. Baseline practical skills deficits were strong predictors of mortality in DS but not in those without DS, even when adjusted for by age, clinical diagnosis of dementia, and other significant predictive variables. This difference could have arisen because participants with DS may have had their baseline functioning already impaired by early cognitive decline which was not recognized by caregivers, and this decline itself increased mortality. Also differing between the DS and non-DS group was the type of depressive symptoms found to be significant predictors of mortality. Baseline physical (rather than behavioral) symptoms of depression including body stress, eating problems, low energy, regressive behaviour, and sleep problem were predictive of increased mortality in participants without DS. In contrast, in participants with DS, behavioural symptoms of depression (anxious, crying spells, fearful, overly sensitive, sadness) were found to be significant predictors on increased mortality. The reason for this discrepancy is not readily apparent.

### 4.3. Study Limitations and Summary

Our study was limited by small sample size, lack of sophisticated imaging, lack of detailed data on causes of death, lack of medication and seizure data throughout the whole study, and lack of individualized and standardized clinical diagnosis. In spite of this, the long follow-up time may provide valuable insights into baseline predictors of serious health outcomes, and may result in further improvements to life expectancy, especially for those with higher mortalities or higher rates of depressive symptoms. In particular, clinicians should take depressive symptoms very seriously, evaluating associated health issues and carefully the necessity for further consultation with specialty services. The use of anticonvulsants for reasons (such as behavioural problems) other than epilepsy should be considered carefully, and perhaps only instituted if there is a lack of response to other interventions. The presence of seizures (early onset and later onset) is always a risk for adverse health outcomes, yet excessive vigilance may also result in decreased autonomous functioning and resultant quality of life.

## Figures and Tables

**Table 1:** Demographics of participants at entry to the study.

	Non-DS			DS			All diagnoses		
Age	Male	Female	Total	Male	Female	Total	Male	Female	Total
<30	24	16	40	13	7	20	37	23	60
30–39	42	32	74	22	19	41	64	51	115
40–49	39	26	65	19	17	36	58	43	101
50–59	21	16	37	8	7	15	29	23	52
60–69	7	10	17	2	2	4	9	12	21
70–79	8	2	10	0	0	0	8	2	10
80–89	1	0	1	0	0	0	1	0	1

Total	142	102	244	64	52	116	206	154	360

Mean	43.17	42.91	43.06	39.73	40.48	40.07	42.1	42.09	42.09

SE	1.18	1.23	0.85	1.27	1.35	0.92	0.91	0.94	0.65

Range	17–83	20–71	17–83	20–61	20–61	20–61	17–83	20–71	17–83

**Table 2:** Summary of key variables entered into the survival model (categorical variables).

Variable	Variable detail	Non-DS		DS		Total	
Deceased	As of July 2011	66	27%	42	36.2%	108	30.0

Dementia	Caregiver reported at baseline	1	0.4%	6	5.2%	7	1.9

Medications	Antipsychotic at baseline	58	23.8%	37	31.9%	95	26.4
	Antipsychotic in any wave	83	34%	49	42.2%	132	36.7
	Antidepressant at baseline	30	12.3%	17	14.7%	47	13.1
	Antidepressant in any wave	56	23%	26	22.4%	82	22.8
	Sedative-hypnotic at baseline	5	2%	6	5.2%	11	3.1
	Sedative-hypnotic in any wave	24	9.8%	14	12.1%	38	10.6
	Anxiolytic at baseline	25	10.2%	11	9.5%	36	10.0
	Anxiolytic in any wave	50	20.5%	26	22.4%	76	21.1
	Anticonvulsant at baseline	66	27%	36	31%	102	28.3
	Anticonvulsant in any wave	81	33.2%	48	41.4%	129	35.8

Seizure history	Seizure history (current or past) at baseline	84	34.4%	14	12.1%	98	27.2
	Seizures (actively) present at baseline	39	16%	6	5.2%	45	12.5
	Seizures reported in any of the four waves	80	32.8%	46	39.7%	126	35
	New seizures reported after baseline	16	6.6%	12	10.3%	28	7.8

*Higher scores indicate greater deficits.

**Higher scores indicate greater yearly increase in deficits between 1995 and 2001.

**Table 3:** Summary of key variables entered into the survival model (continuous variables).

Variable	Variable detail	Non-DS		DS		Total	
		Mean (SE)	Range	Mean (SE)	Range	Mean (SE)	Range
Years followed	As of July 2011	13.26 (0.25)	0.59–16.10	12.26 (0.39)	0.0–16.05	12.93 (0.21)	0–16.10
Age	Baseline	43.06 (0.85)	17–83	40.07 (0.92)	20–61	42.08 (0.66)	17–83
DMR (baseline)*	Practical skills subscale score	2.20 (0.24)	0–16	0.98 (0.22)	0–16	1.81 (0.18)	0–16
Reiss Screen baseline	Depression (Behavioral)	1.30 (0.11)	0–8	0.93 (0.13)	0–7	1.18 (0.08)	0–8
	Depression (Physical)	1.33 (0.10)	0–6	1.44 (0.16)	0–7	1.36 (0.09)	0–7
DMR change per year**	Sum of Cognitive Scores (SCS)	0.33 (0.11)	−5.26–13.14	0.71 (0.18)	−2.12–11.99	0.45 (0.10)	−5.26–13.14
DMR change per year**	Sum of Social Scores (SOS)	0.50 (0.12)	−3.92–8.34	0.82 (0.23)	−6.84–11.60	0.61 (0.11)	−6.84 −11.60

*Higher scores indicate greater deficits.

**Higher scores indicate greater yearly increase in deficits between 1995 and 2001.

**Table 4:** Number (%) of the baseline cohort deceased and the mean age of death as of July 2011.

Sex	Non-DS		DS		All	
	Number (%) deceased	Age of death (SE)	Number (%) deceased	Age of death (SE)	Number (%) deceased	Age of death (SE)
Males	43 (30.3)	56.0 (2.0)	23 (35.9)	61.7 (2.6)	65 (31.9)	59.7 (1.6)
Females	29 (28.4)	61.6 (1.8)	13 (25.0)	60.1 (3.1)	43 (27.6)	58.6 (1.7)
All	66 (27.0)	61.1 (1.7)	42 (36.2)	56.3 (1.3)	108 (30.0)	59.2 (1.2)

**Table 5:** Multivariate Cox regression analysis of mortality, as of July 2011.

	*β* (SE)	Sig.	Hazard ratio (95% CI)
Down Syndrome	−0.34 (1.00)	NS	0.10–5.11
Sex (ref: male)	−0.58 (0.23)	<0.05	0.36–0.88
Baseline age	0.06 (0.01)	<0.0001	1.05–1.09
Baseline DMR practical skills deficits	0.09 (0.03)	<0.005	1.03–1.16
Baseline Reiss behavioral depression	0.25 (0.06)	<0.0005	1.12–1.44
Baseline anticonvulsant use	0.71 (0.33)	<0.05	1.07–3.90
DMR Sum of Social Scores change per year	0.27 (0.05)	<0.0001	1.18–1.45
History of seizure at or before baseline	0.50 (0.24)	<0.05	1.04–2.63
Seizure before or during the study	−0.69 (0.31)	<0.05	0.28–0.91
Baseline age ∗ DS interaction	0.04 (0.02)	0.08	1.00–1.08

**Table 6:** Multivariate Cox regression analysis of mortality, as of July 2011 (Non-DS).

	*β* (SE)	Sig.	Hazard ratio (95% CI)
Baseline age	0.06 (0.01)	<0.0001	1.04–1.08
Baseline Reiss physical depression	0.19 (0.08)	<0.05	1.03–1.41
DMR Sum of Social Scores change per year	0.34 (0.08)	<0.0001	1.21–1.63
History of seizure at or before baseline	0.59 (0.28)	<0.05	1.05–3.09

**Table 7:** Multivariate Cox regression analysis of mortality, as of July 2011 (DS).

	*β* (SE)	Sig.	Hazard ratio (95% CI)
Sex (ref: male)	−0.98 (0.35)	<0.01	0.187–0.75
Baseline age	0.10 (0.02)	<0.0001	1.06–1.15
Baseline DMR practical skills deficits	0.21 (0.07)	<0.005	1.07–1.41
Baseline Reiss behavioral depression	0.27 (0.11)	<0.01	1.07–1.62
DMR Sum of Cognitive Scores change per year	0.32 (0.08)	<0.0005	1.17–1.63
